# Preliminary Immunological Evaluation of Alginate Microparticles Loaded with Recombinant VP2 as an Immersion-Delivered Antigen Against Infectious Pancreatic Necrosis Virus (IPNV) in Salmonids

**DOI:** 10.3390/v18090926

**Published:** 2026-08-22

**Authors:** Yosvania Hevia, Tomás Cancino, Yesseny Vásquez-Martínez, Francisca Tapia, Carla Arancibia, Natalia Riquelme, Iván Valdés, Marcelo Cortez-San Martín

**Affiliations:** 1 Facultad de Química y Biología, Universidad de Santiago de Chile, Santiago 9170022, Chile; yosvania.hevia@usach.cl (Y.H.); yesseny.vasquez@usach.cl (Y.V.-M.);; 2 Laboratorios Veterquimica SA, Santiago 9200000, Chile; tcancino@veterquimica.cl (T.C.); ivaldes@veterquimica.cl (I.V.); 3 Escuela de Medicina, Facultad de Ciencias Médicas, Universidad de Santiago de Chile, Santiago 9170201, Chile; 4 Departamento de Ciencia y Tecnología de Alimentos, Universidad de Santiago de Chile, Santiago 9170022, Chile; carla.arancibia@usach.cl (C.A.); natalia.riquelme@ciq.uchile.cl (N.R.)

**Keywords:** IPNV, VP2, immersion vaccine, recombinant protein, alginate microparticles, salmonids

## Abstract

Infectious pancreatic necrosis (IPN) is a highly contagious viral disease that causes significant economic losses in the global salmon farming industry. Despite the widespread use of vaccines, outbreaks caused by infectious pancreatic necrosis virus (IPNV) continue to occur, even in genetically resistant fish populations. Therefore, the development of alternative vaccination strategies remains a priority for sustainable aquaculture. In this study, we developed a vaccine prototype based on the recombinant IPNV capsid protein VP2 loaded in alginate microparticles for immersion immunization. The VP2 protein was produced using the *E. coli* pET21b+ expression system and subsequently encapsulated in alginate by ionic gelation with calcium chloride. The immunogenic potential of the VP2-MP formulation was evaluated in salmonids by quantifying specific antibody levels and analyzing the transcription of immune-related genes (ifn-α, ifn-γ, mx, and tbet). Fish immunized with VP2-MP showed induction of antiviral immune responses characterized by increased expression of ifn-γ and mx, together with detectable levels of specific antibodies against IPNV-VP2. Overall, these results indicate that alginate microparticles enhance the immunogenicity of the VP2 antigen and support the feasibility of immersion-based vaccination strategies against IPNV in salmonids.

## 1. Introduction

Salmon farming in Chile is primarily based on three species: *Oncorhynchus mykiss* (rainbow trout), *Oncorhynchus kisutch* (coho salmon), and *Salmo salar* (Atlantic salmon), with the latter currently dominating production and export volumes [1]. Over the past decades, the industry has expanded rapidly, becoming a major contributor to the national economy. This growth, however, has been accompanied by an increased incidence of infectious diseases, which remain a key limitation for sustainable production [1,2].

Among viral pathogens, infectious pancreatic necrosis (IPN) continues to be one of the most prevalent and economically relevant diseases in salmonids. It affects fish at different developmental stages and occurs in both freshwater and marine environments [3]. Notably, recent reports indicate a rise in clinical outbreaks during the freshwater phase [4]. The causative agent, infectious pancreatic necrosis virus (IPNV), is a non-enveloped icosahedral virus (~70 nm) with a bi-segmented double-stranded RNA genome encoding at least five proteins [5]. The viral capsid is mainly composed of VP2 and VP3, with VP2 serving as the principal antigenic determinant. This protein contains key neutralizing epitopes and a hypervariable region linked to antigenic diversity and virulence, making it a central target for vaccine design [6,7].

The antiviral immune response in fish involves both innate and adaptive components. Early responses are driven by type I interferons (IFN-α) and downstream antiviral genes such as mx [8,9], while adaptive immunity relies primarily on B cells and the production of immunoglobulin M (IgM) [10]. In the case of IPNV, VP2-specific antibodies have consistently been associated with protection, supporting their role as an important correlate of immunity [11,12].

Recombinant VP2-based antigens have been widely explored using different expression systems. In particular, the production of VP2 alone or in combination with VP2–VP3 complexes in *Escherichia coli* has shown the ability to induce protective immune responses in fish [12,13]. However, while these approaches have yielded encouraging results under controlled experimental conditions, their performance in field settings has been more variable. Protection levels tend to fluctuate and are influenced by several factors, including viral strain, fish age, and the route of vaccine administration [14]. Taken together, these limitations highlight the need for improved antigen delivery strategies that can promote more consistent, robust, and long-lasting immune responses.

Immersion vaccination offers clear practical advantages, particularly for early life stages, as it enables mass immunization without individual handling. Despite this, its effectiveness is frequently limited by low antigen uptake across mucosal surfaces, resulting in weaker immune responses than those induced by injectable vaccines [15,16]. In this context, the ability of antigens to interact with mucosal tissues—especially skin, gills, and their associated lymphoid structures—is a critical determinant of immersion vaccine performance [17].

Micro- and nanotechnology-based approaches have emerged as promising tools to address these limitations. Polymeric microencapsulation systems can enhance antigen stability, facilitate mucosal delivery, and improve uptake by antigen-presenting cells [18,19]. Among these, alginate has attracted particular interest due to its biocompatibility, biodegradability, and ease of formulation. Derived from brown algae such as *Macrocystis pyrifera*, *Ascophyllum nodosum*, and *Laminaria hyperborea*, alginate has been widely used in drug delivery and vaccine applications [20,21,22].

Mucosal tissues play a central role in both pathogen entry and immune activation in fish. The skin, as the largest mucosal surface, represents a key target for immersion vaccination. Previous studies have shown that alginate-based microformulations, particularly when administered orally, can effectively stimulate both innate and humoral responses in salmonids. For example, oral administration of alginate-encapsulated IPNV antigens significantly enhanced the antibody response in Atlantic salmon [23], while other alginate-based oral vaccines have successfully induced protective immune responses against viral and bacterial pathogens in fish [11,22,24]. Although oral and immersion vaccination target different mucosal surfaces, both strategies rely on antigen uptake through mucosal tissues. Therefore, these studies provide a strong rationale for evaluating alginate microencapsulation as an antigen delivery platform for immersion vaccination.

Based on this rationale, the present study aimed to develop an alginate microparticle-based vaccine containing recombinant IPNV VP2 protein for immersion delivery in salmonids. The immunogenicity of this formulation was evaluated by analyzing specific antibody responses and the transcriptional profiles of antiviral immune-related genes.

## 2. Materials and Methods

### 2.1. Cell, Virus, and Culture Conditions

CHSE-214 cells (91041114, ECACC Salisbury, UK) were maintained in Eagle’s Minimum Essential Medium (MEM, Sigma, New York, NY, USA, Cat. 4655) supplemented with 10% fetal bovine serum (FBS, Sigma, New York, NY, USA, Cat. 12106C), 2 mM L-glutamine (Corning, Steuben County, NY, USA, Cat. 25-005-Cl), 10% non-essential amino acids (Gibco, Grand Island, NY, USA, Cat. 11140-035), 100 U/mL penicillin–streptomycin (Gibco, Grand Island, NY, USA, Cat. 15140-122), and 500 μg/mL gentamicin (USBiological, Salem, MA, USA, Cat. 1405-41-0). Cell cultures were incubated at 15 °C, and the culture medium was replaced weekly.

The Chilean IPNV Sp strain isolated in 2022 (Sp lab220114) was used both for amplification of the vp2 open reading frame and for preparation of inactivated viral antigen. CHSE-214 monolayers at approximately 60% confluence were infected with the virus. Once cytopathic effects (CPE) became evident, both the cell layer and the supernatant were collected.

Viral titers were determined by calculating the 50% tissue culture infectious dose (TCID_50_) using the Reed-Muench method [25]. In parallel, viral RNA was quantified by RT-qPCR. Amplification of IPNV genomic segment B was carried out using the primer pair D750F (5′-NTA CAT AGG CAA AAC CAA AGG-3′) and D950R (5′-GGT CTT CWW GTC CCA GTC-3′) following the protocol described by Vázquez et al. [26].

To obtain inactivated virus antigen, a portion of the viral inoculum from infected cells was treated with 37% formalin (Winkler, Frutillar, Chile, Cat. 50-00-0) to reach a final concentration of 0.3% and incubated for seven days at 4 °C. Successful inactivation was verified by inoculating CHSE-214 cells with a 1:100 dilution of the treated virus and monitoring the cultures for the appearance of CPE over four days. The non-inactivated virus served as a positive control.

### 2.2. Amplification of the IPNV VP2 Open Reading Frame

Viral RNA extraction was performed according to the method described by Blake [27]. The vp2 gene coding sequence was subsequently amplified by RT-PCR using primers containing SalI and XhoI restriction sites (Table 1).

For cDNA synthesis, viral RNA was first denatured in the presence of random hexamer primers at 100 °C for 5 min. Reverse transcription reactions contained 10× M-MuLV buffer (New England Biolabs, Ipswich, MA, USA, Cat. B0253S), RNase Out (New England Biolabs, Ipswich, MA, USA, Cat. M0307S), dNTPs (Cytiva, Amersham, UK, Cat. 28-4065-51), and M-MuLV reverse transcriptase (New England Biolabs, Ipswich, MA, USA, Cat. M0253S). The reaction volume was adjusted to 20 µL with nuclease-free water (Corning, Steuben County, NY, USA, Cat. 46-000-CM). Reverse transcription was carried out at 25 °C for 5 min, followed by 42 °C for 60 min and enzyme inactivation at 65 °C for 20 min.

PCR amplification was performed using Paq5000 DNA polymerase (Agilent, Santa Clara, CA, USA, Cat. 600684). The cycling protocol consisted of an initial denaturation step at 95 °C for 2 min, followed by 30 cycles of 95 °C for 20 s, 57.6 °C for 20 s, and 72 °C for 50 s, with a final extension at 72 °C for 5 min. Amplified products were analyzed by electrophoresis on a 0.8% agarose gel stained with GelRed (Biotium, Fremont, CA, USA, Cat. 41003) and visualized under UV illumination.

### 2.3. Cloning, Expression, and Purification of Recombinant VP2 Protein

The amplified vp2 fragment and the pET21b+ vector (Sigma, USA) were digested with SalI (New England Biolabs, Ipswich, MA, USA, Cat. R0138S) and XhoI (New England Biolabs, Ipswich, MA, USA, Cat. R0146S) in order to clone the vp2 fragment and to obtain the recombinant protein with His-tag in C-terminus Ligation was performed using T4 DNA ligase (Sigma, USA) according to the manufacturer’s instructions.

The resulting plasmid constructs were introduced into competent *E. coli* DH5α cells (1 × 10^8^ CFU/µg DNA) by heat-shock transformation. Transformed cells were plated on LB agar (Sigma-Aldrich, Burlington, MA, USA, Cat. L3147) containing ampicillin (100 µg/mL; Sigma-Aldrich, Burlington, MA, USA, Cat. A5354) and incubated overnight. Plasmid DNA from selected colonies was purified using the E.Z.N.A. Plasmid DNA Mini Kit I (Omega, New York, NY, USA, Cat. D6942-01), and the correct insertion and sequence of the VP2 ORF was confirmed by Sanger sequencing (Macrogen).

For recombinant protein production, the verified plasmid was transformed into *E. coli* BL21(DE3) cells. Cultures were grown in LB medium supplemented with ampicillin until an OD600 of approximately 0.7 was reached. Protein expression was induced with 1 mM IPTG (Merck, Darmstadt, Germany, Cat. 367-93-1), and incubation was continued at 15 °C with shaking (200 rpm) for 24 h.

Proteins separated by SDS-PAGE were visualized with Coomassie Brilliant Blue (Sigma-Aldrich, Burlington, MA, USA, Cat. 1.15444). Expression of VP2 was assessed by SDS-PAGE and Western blot. Detection was performed using an HRP-conjugated anti-His antibody (Miltenyi Biotec, Bergisch Gladbach, Germany, Cat. 130-092-785).

The bacterial pellet containing recombinant VP2 protein was solubilized in 8 M urea (Sigma-Aldrich, Burlington, MA, USA, Cat. U5378) prepared in 0.1 M Tris-HCl (pH 7.6). The solubilized protein was subsequently dialyzed against PBS supplemented with 1 mM phenylmethylsulfonyl fluoride (PMSF; Merck, Darmstadt, Germany, Cat. 10837091001) using a regenerated cellulose membrane (Sigma-Aldrich, Burlington, MA, USA, Cat. D9527). Recombinant protein identity was confirmed by MALDI-MS/MS peptide mass fingerprinting using a MALDI-TOF MS Autoflex Speed (Bruker Daltonics, Bremen, Germany) at the Proteomics and Metabolomics Unit, University of La Frontera, Chile. For the analysis, the prominent band observed in the SDS-PAGE gel was excised, digested with trypsin (Promega, Tokyo, Japan, Cat. V5280), and subsequently analyzed by mass spectrometry. Peptide identification confidence was supported by Mascot scores and spectral quality, with high-confidence peptides defined by higher scores and/or longer peptide length.

### 2.4. Preparation of VP2-Loaded Alginate Microparticles

Alginate microparticles were prepared using an ionic gelation method with minor modifications [22]. Briefly, the recombinant VP2 protein solution obtained in the previous step (0.5 mg/mL) was mixed with 1.5% (*w*/*v*) sodium alginate (Sigma-Aldrich, Burlington, MA, USA, Cat. W201502). Control microparticles were generated by replacing the protein solution with PBS (pH 7.4).

The alginate–protein mixture was emulsified in liquid paraffin containing 0.5% Span 80 (Sigma-Aldrich, Burlington, MA, USA, Cat. S6760) using an Omni TH homogenizer operating at 20,000 rpm. Cross-linking was initiated by adding calcium chloride solution (8% *w*/*v*; Sigma-Aldrich, Burlington, MA, USA, Cat. C1016). Microparticles formed during this process were recovered by centrifugation (9000 rpm, 10 min), washed with ethanol solutions, freeze-dried (−55 °C, 0.040 mbar), and stored at 4 °C until further use.

### 2.5. Microparticle Characterization

Microparticles were resuspended in distilled water prior to analysis. Their hydrodynamic diameter, polydispersity index (PDI), and zeta potential were measured by dynamic light scattering using a Zetasizer Nano ZS90 (Malvern Instruments, Worcestershire, UK). Particle morphology and size were further evaluated by transmission electron microscopy (TEM, Hitachi, Tokyo, Japan) and scanning electron microscopy (SEM, Zeiss EVO I MA10). For TEM, a drop of the microparticle suspension was deposited onto a copper grid and air-dried at room temperature. For SEM, the microparticle suspension was deposited onto a sample holder, air-dried, and sputter-coated with gold.

Encapsulation efficiency (EE) and loading capacity (LC) were calculated based on the amount of protein released from microparticles after incubation in sodium citrate buffer (Sigma-Aldrich, Burlington, MA, USA, Cat. S4641).

### 2.6. Fish Husbandry and Immunization Assay

Atlantic salmon (*Salmo salar*) (12 ± 3 g) were maintained in 200 L freshwater tanks under recirculating conditions at 16 °C, with dissolved oxygen levels ranging from 5.8 to 9 mg/L, pH 6.6–6.9, and salinity of 3–5‰. Fish were fed daily and fasted for 24 h prior to vaccination. Prior to the experiment, fish were screened for infectious pancreatic necrosis virus (IPNV) according to OIE guidelines [28] to confirm their pathogen-free status. All experimental procedures performed in this study were reviewed and approved by the Institutional Committee for the Care and Use of Laboratory Animals (CICUAL) of Veterquímica.

**Table 1:** Primers used for the amplification of the vp2 gene and for the evaluation of the immune response through transcript analysis.

Gen	Foward Sequence	Reverse Sequence	Reference
VP2	ACGCGTCGACATGAACACAAACAAGGCA	CGGCTCGAGCCGAATTCCTCTGACTA	-
IFNα	AAAACTGTTTGATGGGAATATGAAA	CGTTTCAGTCTCCTCTCAGGTT	[11]
IFNγ	TTCAGGAGACCCAGAAACACTAC	TAATGAACTCGGACAGAGCCTTC	[29]
Mx	TGATCGATAAAGTGACTGCATTCA	TGAGACGAACTCCGCTTTTTCA	[30]
Tbet	GGTAACATGCCAGGGAACAGGA	TGGTCTATTTTTAGCTGGGTGATGTCTG	[31]
18s	TGTGCCGCTAGAGGTGAAATT	GCAAATGCTTTCGCTTTCG	[32]

A total of 240 fish were randomly allocated into four experimental groups, each comprising two biological replicates of 30 fish (60 fish per treatment): (i) microencapsulated recombinant VP2 (VP2-MP), (ii) inactivated IPNV (IPNV), (iii) empty alginate microparticles (Al-MP), and (iv) phosphate-buffered saline (PBS) as a negative control. Following vaccination, each treatment group was maintained in a separate tank, with both biological replicates housed together under identical environmental conditions throughout the experimental period.

Immunization was performed by immersion. Briefly, 30 fish were immersed in 20 L of water (35 g fish/L) containing 20 mL of the corresponding formulation, resulting in a final concentration of 0.54 mg/mL recombinant VP2 protein or 1 × 10^8^ TCID_50_/mL of inactivated virus. Fish were maintained under continuous aeration for 6 h and subsequently returned to their respective pre-assigned holding tanks. Control groups were treated identically.

### 2.7. Sample Collection

Serum and anterior kidney samples were collected from five fish per group at 0, 1, 3, 18, and 30 days post-vaccination (dpv). Fish were euthanized under anesthesia with 120 mg/L p-aminobenzoate (Veterquímica, Santiago Región Metropolitana, Chile).

Blood samples were collected from the caudal vein and allowed to clot at room temperature for 2 h, followed by incubation at 4 °C for 4 h. Samples were centrifuged at 1500× *g* for 10 min at 4 °C, and serum was recovered and stored at 4 °C until use.

Anterior kidney samples were preserved in RNAlater (Thermo Scientific, Waltham, MA, USA, Cat. AM7020) and stored at −20 °C until further processing.

### 2.8. ELISA for Quantification of Anti-IPNV IgM

Specific anti-IPNV IgM antibodies were measured by indirect ELISA. Plates were coated overnight with the synthetic peptide VQ-Pep_IPN-001 (Biotina-KPETGPASIPDDITDFSSDLPTSKAW, designed and validated by Veterquimica S.A.) containing B-cell epitopes from the IPNV capsid. After blocking with 2% FBS in PBS, serum samples were added in serial dilutions (1:4–1:256).

Detection was carried out using a mouse anti-salmonid IgM monoclonal antibody (RayBiotech, Peachtree Corners, GA, USA) followed by an HRP-conjugated mouse anti-IgG secondary antibody (KPL, San Diego, CA, USA). Color development was achieved using TMB substrate, and absorbance was measured at 450 nm with a microplate reader (Sunrise, Tecan, Grodig Austria).

### 2.9. Gene Expression Analysis by RT-qPCR

Total RNA was extracted from anterior kidney tissue using TRIzol reagent (Invitrogen, Carlsbad, CA, USA, Cat. 15596026). Complementary DNA (cDNA) was synthesized using M-MLV reverse transcriptase (Promega, Madison, WI, USA, Cat. M1705).

Gene expression analysis for ifn-α, ifn-γ, mx, and tbet was performed by RT-qPCR using a LightCycler^®^ 96 system (Roche, Basel, Switzerland) and Takyon ROX SYBR MasterMix (Eurogentec, Ougrée, Belgium, Cat. UF-RSMT-B0701). Relative expression levels were normalized against 18S rRNA and calculated using the 2^−ΔΔCt^ method.

### 2.10. Statistical Analysis

Statistical analyses were performed using the Kruskal–Wallis test followed by Dunn’s multiple comparison test with Bonferroni correction to adjust for multiple comparisons. Comparisons were performed both among treatment groups at each sampling time and among sampling times within each treatment. For ELISA analyses, each serum dilution (1:32, 1:64, 1:128, and 1:256) was analyzed independently using the same statistical approach. All analyses were conducted using RStudio version 4.5.1 (2025). Differences were considered statistically significant when *p* < 0.05.

## 3. Results

### 3.1. Recombinant Expression of the VP2 Antigenic Protein in Escherichia coli

**Figure 1:**
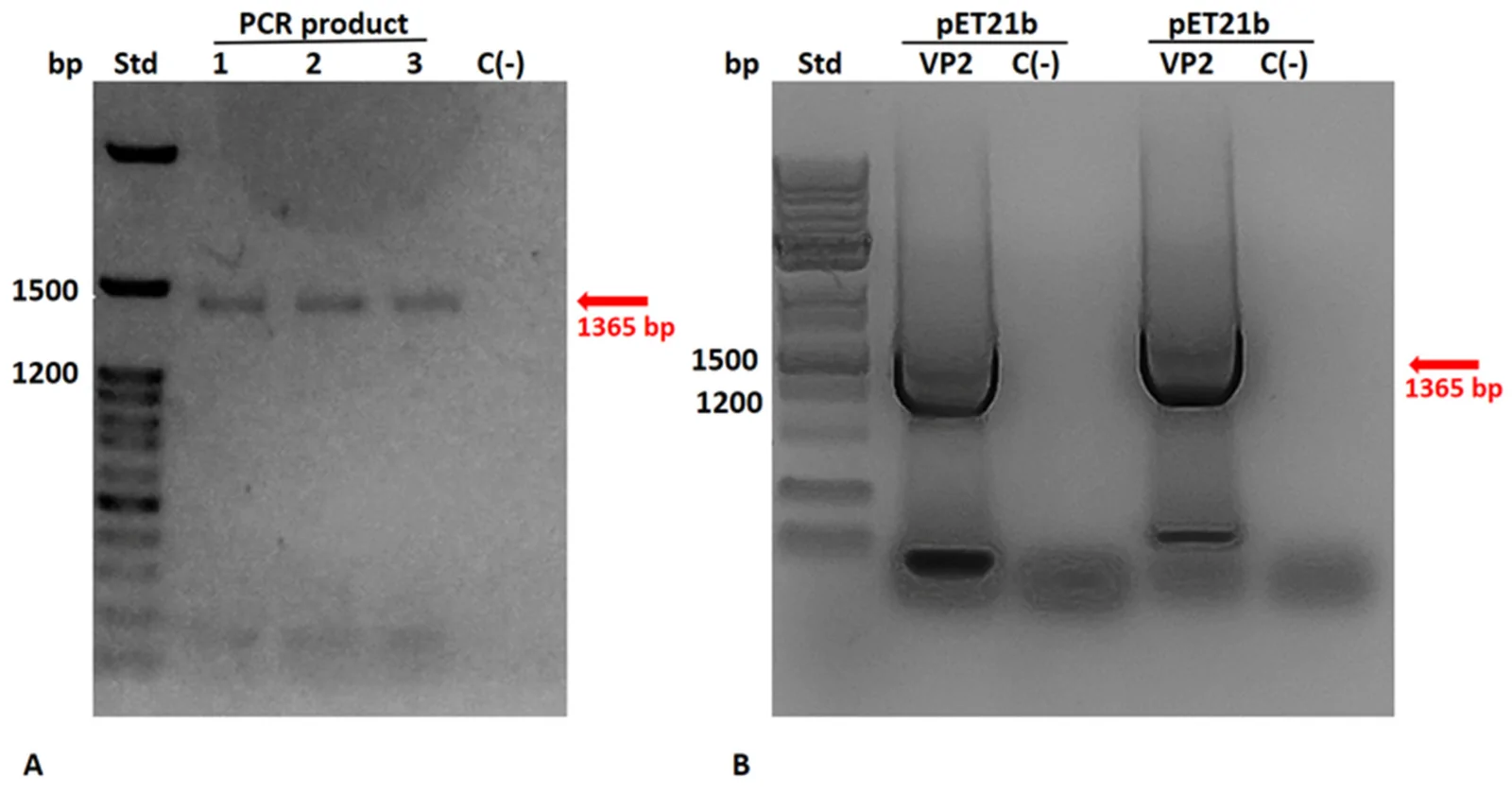
Agarose gel electrophoresis of PCR products. (**A**) Amplification of the complete VP2 open reading frame (ORF) using specific primers (lanes 1–3). (**B**) Amplification from pET21b(+)/VP2 using primer pairs VP2_SalI_Fw/VP2_XhoI_Rv (lane 2) and VP2_SalI_Fw/T7 terminator (lane 4).

CHSE-214 cells were infected with IPNV at a multiplicity of infection (MOI) of 0.1 to amplify viral stocks, reaching a viral load of 1.2 × 10^7^ RNA copies/mL at 4 days post-infection, as determined by RT-qPCR targeting genome segment B. Viral RNA was used as template to amplify the complete VP2 ORF by RT-PCR, yielding a product of approximately 1.5 kb, consistent with the expected size (Figure 1A).

**Figure A1:**
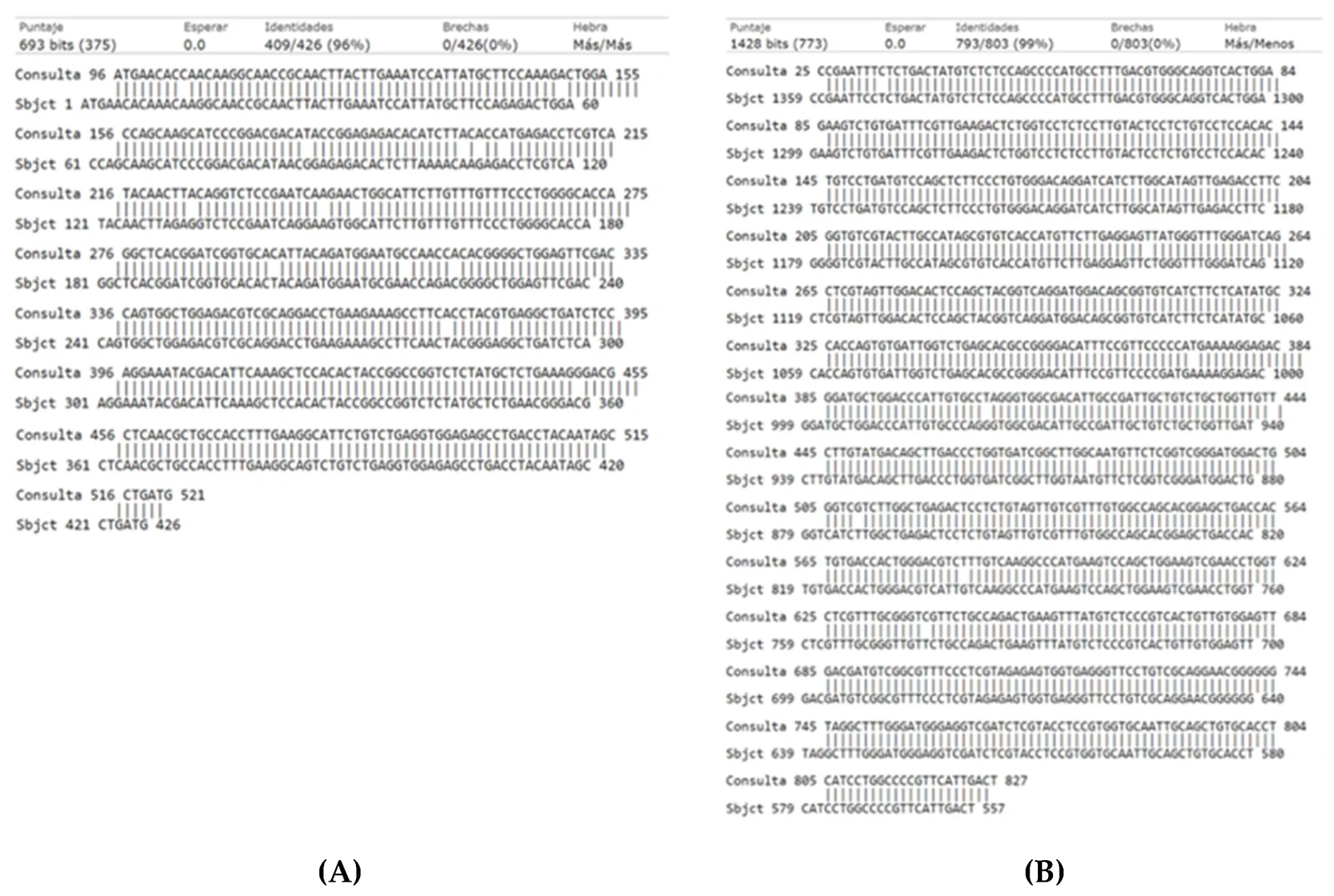
Sequence alignment of the amplified VP2 open reading frame (ORF). The obtained amplicon (~1.5 kb) was analyzed using BLASTn against reference IPNV sequences available in public databases. The alignment confirmed the identity of the amplified fragment as VP2, showing high sequence similarity with reported IPNV strains (AF342728.1). Conserved regions and nucleotide identity across the alignment are indicated. (**A**) Forward sequence alignment. (**B**) Reverse sequence alignment.

The purified PCR product was cloned into pET21b+ and transformed into *E. coli* BL21. The presence and orientation of the insert were verified by PCR (Figure 1B). Sanger sequencing showed 96% nucleotide identity with the vp2 gene of IPNV strain Sp (Figure A1), confirming that the cloned fragment corresponded to the expected viral gene.

**Figure 2:**
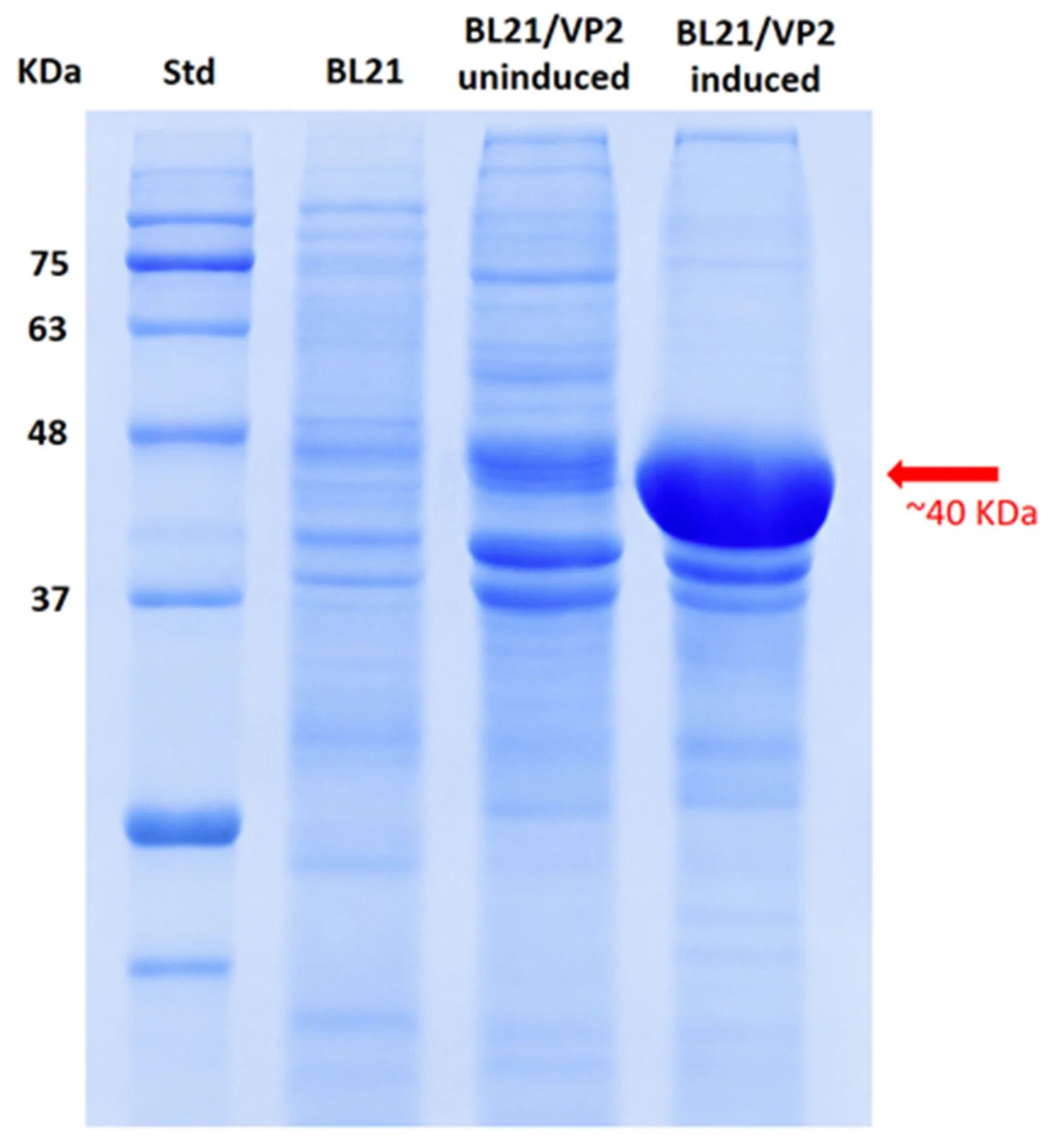
SDS–polyacrylamide gel electrophoresis (SDS–PAGE) of the insoluble protein fraction from *E. coli* BL21 cultures. Lane 1: non-transformed cells; lane 2: cells transformed with pET21b(+)/VP2 without IPTG induction; lane 3: cells transformed with pET21b(+)/VP2 after IPTG induction. Gel stained with Coomassie Brilliant Blue.

**Figure 3:**
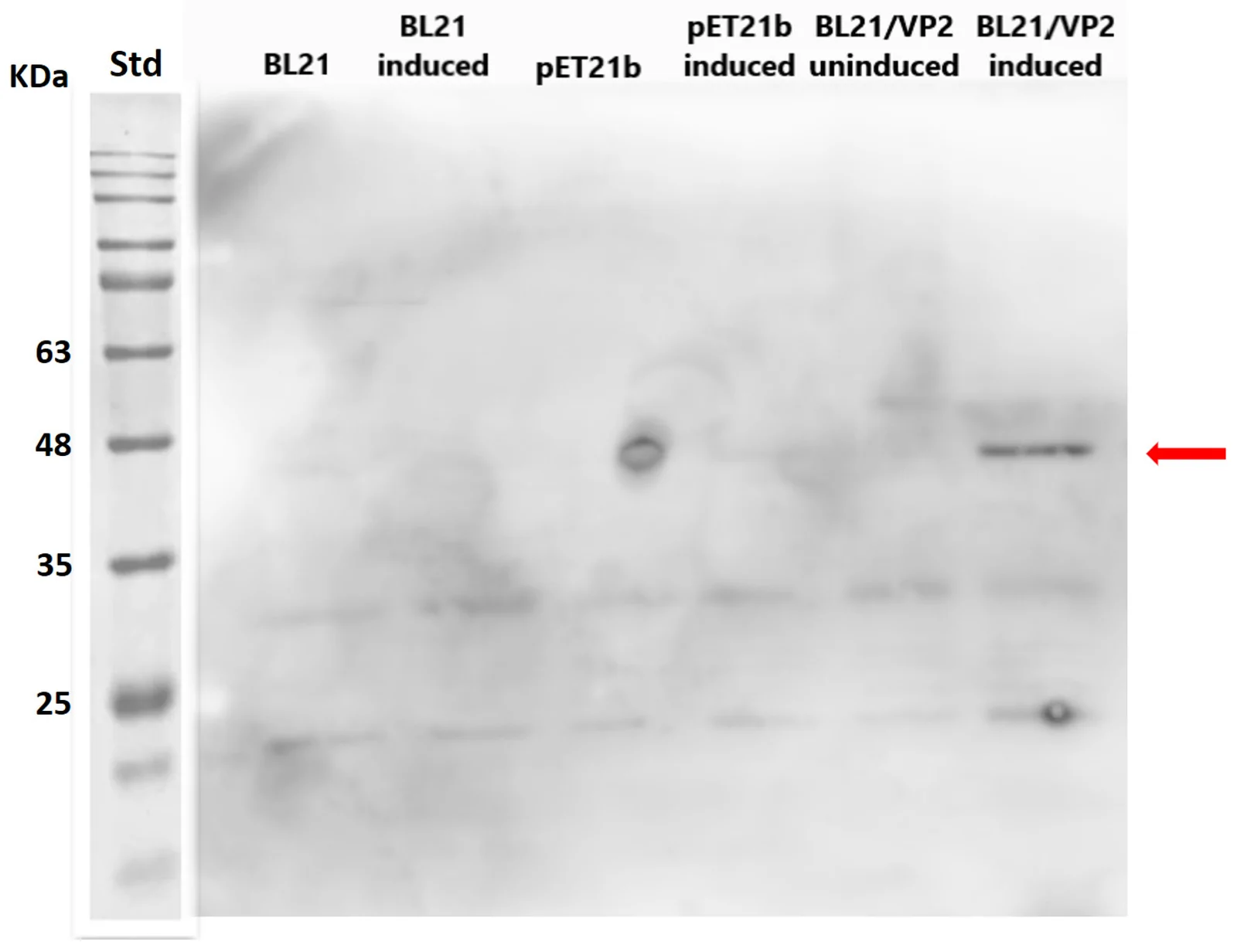
Western blot analysis of recombinant IPNV VP2 expression in *E. coli* BL21(DE3). Whole-cell protein extracts were separated by SDS-PAGE, transferred onto a PVDF membrane, and probed with anti-His monoclonal antibody. Lane Std, standard or molecular weight marker (kDa); lane 1, non-transformed BL21(DE3); lane 2, IPTG-induced BL21(DE3); lane 3, BL21(DE3) harboring the empty pET21b vector (uninduced); lane 4, IPTG-induced BL21(DE3) harboring the empty pET21b vector; lane 5, BL21(DE3) harboring pET21b-VP2 (uninduced); lane 6, IPTG-induced BL21(DE3) harboring pET21b-VP2. The immunoreactive band corresponding to recombinant VP2 (~40 kDa) is indicated by the red arrow.

Following IPTG induction, recombinant VP2 expression was evaluated by SDS-PAGE. A prominent protein band between 40 and 48 kDa was detected in induced cultures, exhibiting an apparent molecular weight lower than the theoretical expected mass of VP2 (~54 kDa), whereas this band was absent in non-induced and control cultures (Figure 2). Western blot analysis using an anti-His monoclonal antibody confirmed the expression of the His-tagged VP2 protein, mainly ~40 kDa, with the highest abundance in the short version (Figure 3).

**Figure 4:**
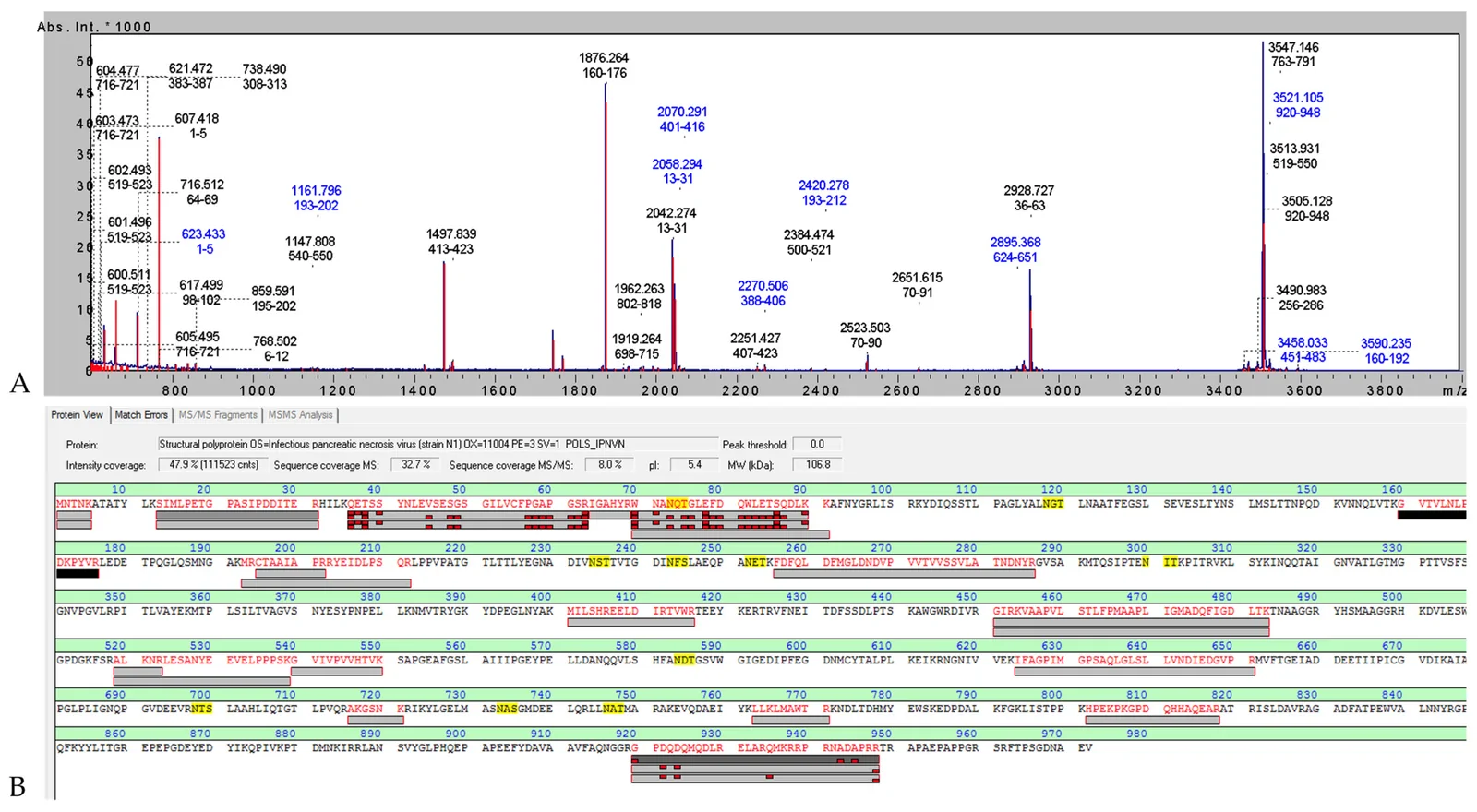
MALDI-MS/MS analysis of recombinant VP2 protein. (**A**) Representative MALDI-TOF mass spectrum of tryptic peptides. (**B**) Sequence coverage map showing identified peptides mapped onto the VP2 protein sequence (38.6% coverage) as aminoacidic residues marked in Red.

MALDI-MS/MS analysis of the recombinant protein with ~40 kDa enabled the identification of 25 tryptic peptides, of which 13 specifically matched the IPNV VP2 sequence, resulting in an overall sequence coverage of 38.6%. The detected peptides were distributed along the protein sequence, providing strong evidence supporting the correct identity of the recombinant VP2 antigen (Figure 4, Table A1). Total protein concentration prior to purification was 1.31 mg/mL, of which approximately 60% corresponded to VP2.

### 3.2. Formulation and Physicochemical Characterization of VP2-Loaded Alginate Microparticles

Alginate microparticles loaded with recombinant VP2 protein produced in *E. coli* were successfully obtained using an ionic gelation method with calcium chloride as the crosslinking agent. In parallel, empty alginate microparticles were prepared using the same procedure, substituting PBS for VP2, and used as vehicle controls in subsequent assays.

**Figure 5:**
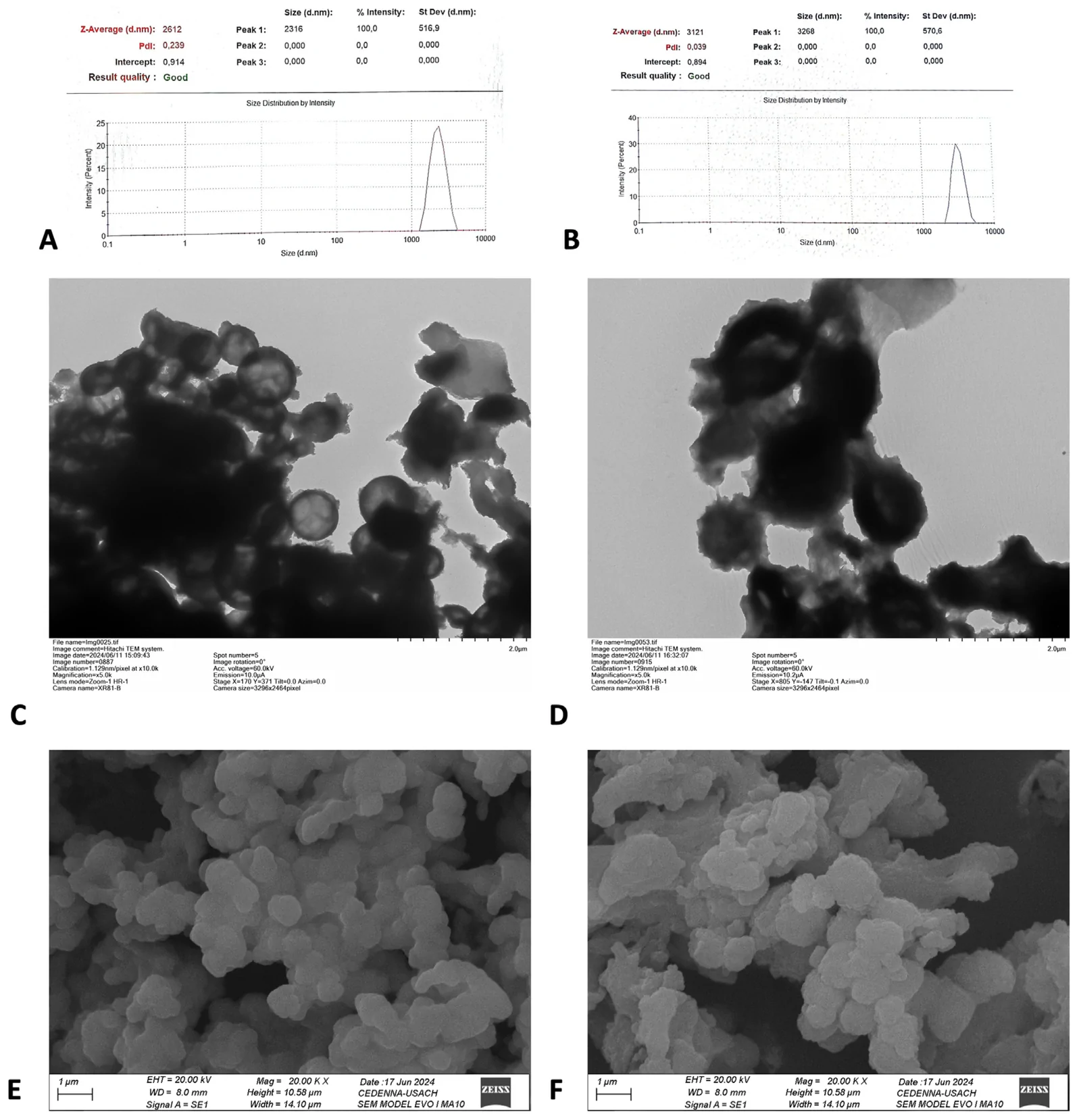
Physicochemical characterization of alginate microparticles. Microparticles loaded with recombinant VP2 protein (**A**,**C**,**E**) and empty microparticles (**B**,**D**,**F**) were analyzed by dynamic light scattering (DLS) (**A**,**B**), transmission electron microscopy (TEM) (**C**,**D**), and scanning electron microscopy (SEM) (**E**,**F**).

The characterization of both VP2-loaded and empty alginate microcapsules included particle size, size distribution, and surface charge analyses. Dynamic light scattering (DLS) revealed hydrodynamic diameters ranging from 3121 to 4443 nm for empty microparticles and from 2316 to 3518 nm for VP2-loaded microparticles, with polydispersity index (PDI) values of 0.039–0.371 and 0.239–0.606, respectively (Figure 5A,B). In contrast, TEM analysis showed significantly smaller particle sizes, ranging from 1000 to 1500 nm for empty microparticles and from 500 to 800 nm for VP2-loaded microparticles (Figure 5C,D). In both formulations, amorphous material compatible with alginate residues was observed, which may explain the discrepancies between DLS and TEM measurements and the variability in PDI values.

TEM images revealed that VP2-loaded microparticles exhibited a uniform spherical morphology with well-defined edges and a clear electron-dense contrast between the core and the shell (Figure 5C), suggesting effective encapsulation of VP2. Conversely, empty microparticles displayed less uniform spherical shapes and lacked a clear electron-dense differentiation (Figure 5D). Scanning electron microscopy (SEM) analysis did not reveal significant morphological differences between loaded and empty microparticles, with both formulations showing irregular, spherical particles with rough surfaces (Figure 5E,F).

Surface charge analysis by zeta potential showed values of −17.3 ± 0.6 mV for VP2-loaded microparticles and −24.6 ± 0.5 mV for empty microparticles. These values are consistent with moderate colloidal stability and a low tendency toward aggregation.

**Figure 6:**
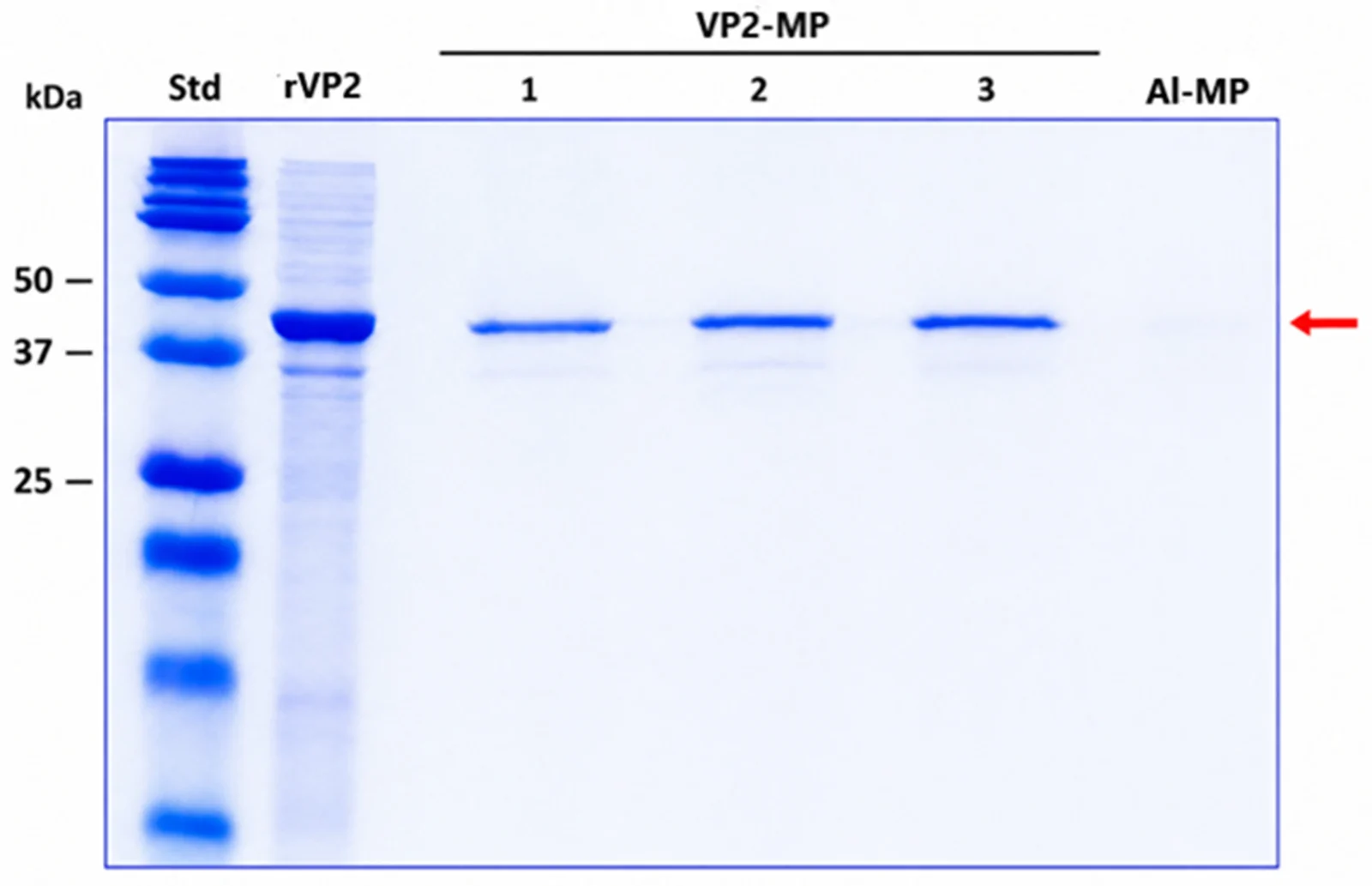
SDS–PAGE analysis of VP2 protein integrity following release from microencapsulated formulations by sodium citrate treatment. Line Std corresponds to the standard (molecular weight marker); lane rVP2 corresponds to purified recombinant VP2 used as a positive control; lanes 1–3 correspond to VP2 released from three independent VP2-MP batches; lane 4 shows the non-encapsulated recombinant VP2 protein used as a negative control. The blue colored band corresponding to recombinant VP2 (~40 kDa) is indicated by the red arrow. Gel stained with Coomassie Brilliant Blue.

Finally, Table 2 summarizes the encapsulation efficiency and loading capacity values obtained from three independent VP2-MP vaccine batches, with encapsulation efficiencies ranging from 41.3% to 46.9% and loading capacities near 13%. The integrity of the released VP2 protein was evaluated by SDS–PAGE (Figure 6). In the lanes corresponding to VP2-loaded microparticles, an intense band close to 40 kDa was observed, consistent with the molecular weight of the recombinant VP2 used for encapsulation, whereas no bands were detected in the empty microparticle control. These results indicate that the protein maintained its integrity throughout encapsulation and release.

### 3.3. Evaluation of the Immune Response Induced by the Microencapsulated Recombinant Vaccine Compared with a Naked Inactivated Virus Vaccine, Administered by Immersion in Salmo salar

#### 3.3.1. Humoral Immune Response Induced by the VP2-MP Vaccine

**Figure 7:**
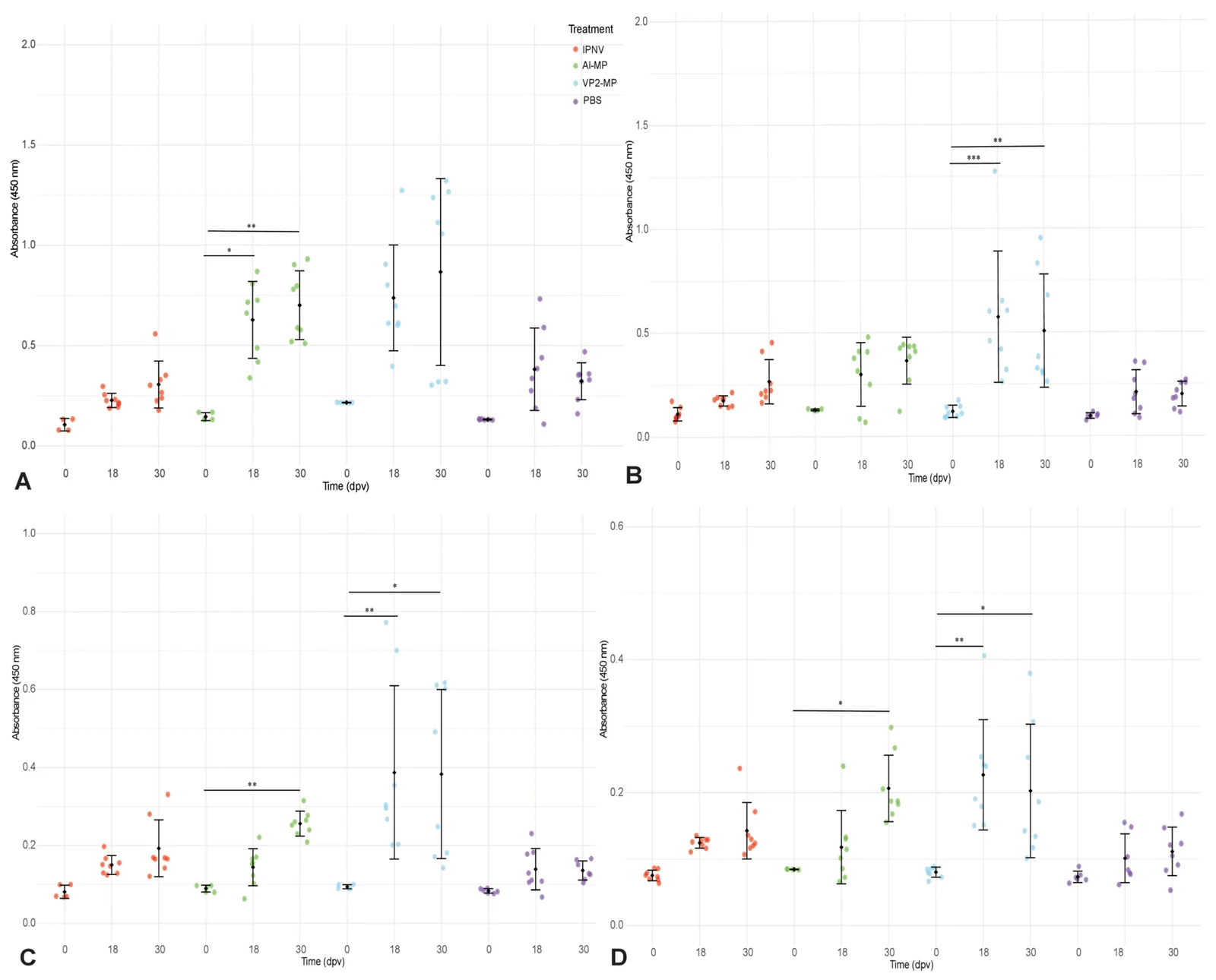
Serum IgM antibody response in *Salmo salar* following immersion immunization. Absorbance values were determined by ELISA at serum dilutions of 1:32 (panel (**A**)), 1:64 (panel (**B**)), 1:128 (panel (**C**)), and 1:256 (panel (**D**)). Statistical differences among groups were analyzed using the Kruskal–Wallis test followed by Dunn’s multiple comparison test with Bonferroni correction. Significance levels are indicated as * *p* < 0.05, ** *p* < 0.01, and *** *p* < 0.001.

To evaluate the humoral immune response induced by the microencapsulated vaccine prototype VP2-MP, specific IgM levels were quantified by indirect ELISA in *S. salar* at 0, 18, and 30 days post-immunization (dpi), using four serum dilutions (1:32, 1:64, 1:128, and 1:256) (Figure 7). Overall, microparticle-based treatments, particularly VP2-MP, showed a progressive increase in antibody response over time compared to IPNV and PBS controls.

At the 1:32 dilution (Figure 7A), the highest absorbance values were observed. The VP2-MP group exhibited a clear increase from 18 dpi, reaching the highest values at 30 dpi. But, at this time point, it is interesting to note that most individuals clustered within high absorbance ranges, although a subset of low responders was also identified. The dispersion observed at both 18 and 30 dpi indicates inter-individual variability. In contrast, the Al-MP treatment showed greater heterogeneity, with some individuals reaching high values (Abs. 0.5 to 1) but without a clear trend across the group. PBS and IPNV remained within low to moderate absorbance ranges (below Abs. 0.5). Although statistically significant differences were detected for Al-MP at 18 and 30 dpi, VP2-MP consistently showed higher responses, suggesting a greater capacity to induce detectable antibodies at low dilutions.

As serum dilution increased (Figure 7B,C; 1:64 and 1:128, respectively), absorbance decreased as expected; however, the relative pattern among treatments remained unchanged. VP2-MP continued to show higher responses, with a significant increase at 18 dpi that persisted at 30 dpi. Although no statistically significant differences were observed between the VP2-MP and PBS groups, likely due to the variability within the VP2-MP group, the data suggest a trend toward higher antibody levels in vaccinated fish compared with the PBS group at each time point analyzed. A bimodal distribution pattern was observed at 30 dpi, characterized by two subpopulations of fish: one with high absorbance values and another with more moderate levels. In the Al-MP group, absorbance values remained variable and generally comparable to those of PBS and IPNV controls, although a significant increase was observed at 30 dpi at the 1:128 dilution.

At the highest dilution (1:256; Figure 7D), VP2-MP maintained detectable antibody levels. A significant increase was observed at 18 dpi, indicating an early response to the encapsulated antigen. At 30 dpi, both microparticle-based treatments showed significant increases; however, VP2-MP reached the highest values, approaching an absorbance of 1 in the highest-responding individuals. In contrast, IPNV and PBS displayed low values with limited variability.

At 0 dpi, low and homogeneous baseline values were observed, with no significant differences between treatments across all dilutions, confirming the initial comparability of the experimental groups. Taken together, the data show that VP2-MP induces higher antibody levels that remain detectable even at increased serum dilutions. This pattern is consistent with improved antigen performance, likely associated with enhanced antigen availability and presentation to the immune system.

#### 3.3.2. Modulation of Antiviral Immune-Related Genes Following VP2-MP Immunization

As part of the analysis of the immune response induced by the different treatments, the relative expression of antiviral immune-related genes (ifn-α, mx, ifn-γ, and t-bet) was evaluated in the anterior kidney of *S. salar* at 0, 24, and 72 h post-immunization. Statistical analysis revealed significant effects of treatment and time (*p* < 0.05), particularly at 24 and 72 h, indicating that gene expression patterns varied with both stimulus and time post-immunization.

**Figure 8:**
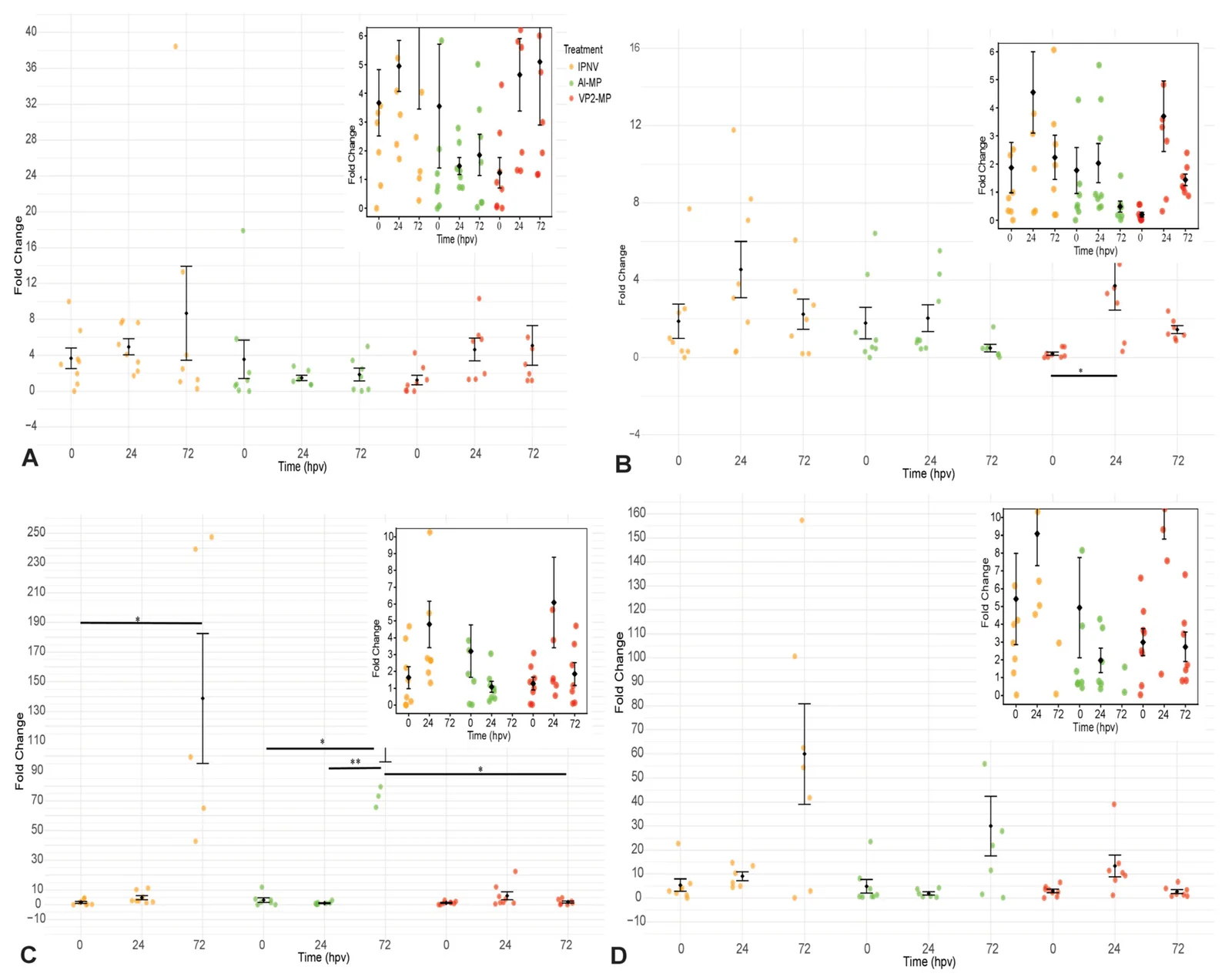
Relative expression of immune-related genes in the anterior kidney of *Salmo salar* analyzed by RT-qPCR. Target genes included ifn-α (panel (**A**)), mx (panel (**B**)), t-bet (panel (**C**)), and ifn-γ (panel (**D**)). Differences among experimental groups were evaluated using the Kruskal–Wallis test followed by Dunn’s multiple comparison test with Bonferroni correction. Significance levels are indicated as * *p* < 0.05 and ** *p* < 0.01.

Regarding the innate immune response, ifn-α expression (Figure 8A) increased markedly in the IPNV group, especially at 72 h, reaching high fold-change values accompanied by substantial inter-individual variability. In contrast, VP2-MP induced a more moderate response, with a peak at 24 h (approximately 3–5-fold change) followed by stabilization at 72 h, whereas Al-MP showed a low and transient response, generally remaining within 0.5–3-fold change. A similar pattern was observed for mx expression (Figure 8B), which showed significant induction in the VP2-MP group at 24 h (approximately 3–6-fold change). In the IPNV group, mx expression reached high levels (10–12-fold change) but showed greater dispersion among individuals, whereas Al-MP remained at low levels across the evaluated time points (approximately 0.5–2-fold change).

Analysis of adaptive immune-related genes showed that ifn-γ (Figure 8D) and t-bet (Figure 8C) followed similar expression patterns. The IPNV group showed the highest ifn-γ levels, particularly at 72 h, with elevated fold changes and pronounced variability. By comparison, VP2-MP exhibited a moderate and sustained increase in ifn-γ expression (approximately 4–8-fold change) with lower dispersion and a more gradual temporal pattern. Similarly, t-bet expression was higher in IPNV (10–60-fold change) at 72 h but with high variability, whereas VP2-MP showed earlier induction at 24 h followed by stabilization. The Al-MP group showed low to moderate expression levels during the first 24 h, followed by a marked increase at 72 h. The parallel trends observed between t-bet and ifn-γ are consistent with coordinated activation of this pathway, which appeared more uniform in VP2-MP than in IPNV.

Two main expression profiles were identified. The IPNV treatment was associated with high fold-change values, marked inter-individual variability, and a peak response at 72 h. MP-VP2, however, showed intermediate fold-change values (approximately 3–10-fold), lower variability, and earlier activation at 24 h. The Al-MP treatment exhibited limited activation across all evaluated genes specially at 24 h post immunization.

## 4. Discussion

IPN remains one of the most economically relevant viral diseases affecting salmon aquaculture in Chile. The endemic nature of IPNV, its high prevalence across developmental stages, and its capacity to establish persistent infections with intermittent shedding create favorable conditions for horizontal transmission and recurrent outbreaks [4]. Despite the availability of commercial IPNV vaccines in several countries, outbreaks still occur, and protective efficacy under field conditions can be variable [14,33]. In this context, the present study explored an alternative vaccination strategy based on a recombinant VP2 antigen formulated in alginate microparticles for immersion administration, aiming to improve feasibility for early life stages and enhance the quality and kinetics of immune stimulation at mucosal interfaces.

### 4.1. Recombinant VP2 Production and Antigen Identity

Efficient production of recombinant antigens is a key step for protein-based vaccine development. Previous studies have reported successful VP2 expression using different plasmids, induction regimes, and incubation temperatures [12,13,34]. In the present study, the pET21b+/BL21(DE3) expression system enabled robust VP2 overexpression, consistent with the widespread use of *E. coli* as a platform for recombinant biopharmaceutical production [35,36]. Protein expression was achieved using a modified protocol based on the methodology described by Tamer et al. [13]. SDS-PAGE (Figure 2) and Western blot (Figure 3) analyses confirmed VP2 expression; however, the predominant band showed an apparent molecular weight of approximately 40 kDa, lower than the theoretical VP2 mass (~54 kDa). Similar findings have previously been associated with proteolysis or partial truncation of VP2 [5,37]. 

The possibility that the observed difference in apparent molecular mass was related to the absence of glycosylation in the bacterial expression system was also considered. VP2 of infectious pancreatic necrosis virus is a glycosylated protein, with evidence supporting the presence of O-linked glycosylation [13,37]. Thus, the absence of this post-translational modification in *E. coli* could contribute to differences in the apparent molecular mass and electrophoretic mobility of the recombinant protein during SDS-PAGE. Alternatively, the anomalous migration observed here may reflect expression system-dependent effects, including differences in protein conformation and SDS-binding efficiency, which have been reported for recombinant viral structural proteins produced in *E. coli* [38]. Based on the present data, these possibilities cannot be distinguished solely by SDS-PAGE, and therefore the difference between the predicted and experimentally observed molecular mass should be interpreted cautiously. Expression of VP2 in *E. coli* may also affect protein folding, solubility, aggregation state, or accessibility of the affinity tag, potentially contributing to the very low recovery observed during purification attempts using Ni-NTA affinity chromatography. Consequently, because Ni-NTA purification yielded insufficient amounts of protein for subsequent analyses, the dialyzed protein preparation was used for peptide mass fingerprinting and further characterization.

To further confirm protein identity under these conditions, MALDI-MS/MS was used as an orthogonal approach. MALDI-based peptide identification is widely applied for rapid and reliable protein confirmation, with confidence primarily supported by the number, specificity, and scores of the matched peptides [39,40]. In our dataset, analysis of 25 tryptic peptides identified 13 VP2-specific peptides matching the IPNV VP2 sequence, resulting in 38.6% sequence coverage. This level of coverage is generally considered robust for viral structural proteins, where tryptic digestion efficiency and ionization biases often limit complete sequence representation [41,42]. Notably, sequence coverage was predominantly supported by medium- to high-mass peptides, which are typically associated with higher identification confidence and improved structural representation. In addition, the distribution of peptides along the VP2 sequence, including N-terminal and central regions, reinforces the robustness of protein identification [40,41]. The relatively low proportion of short peptides further reduces the likelihood of false-positive matches and contributes to overall confidence in peptide assignment [39,40]. Together, immunoblotting and mass spectrometry provide convergent and complementary evidence supporting the identity of the expressed VP2 antigen that migrate around 40 kDa.

Overall, these results validate the successful production of an immunologically recognizable recombinant VP2 protein, although future strategies should focus on improving its solubility and folding to enhance the recovery of a structurally optimized antigen with potential vaccine plataforms.

### 4.2. Alginate Formulation and Implications of Physicochemical Properties

Alginate is a well-established biopolymer for drug and vaccine delivery due to its biocompatibility, mucoadhesion, and mild gelation conditions. Crosslinking with divalent cations—particularly calcium, given its safety and broad biomedical use—facilitates the formation of stable gel matrices suitable for encapsulating biomolecules [21,43]. Ionic gelation is therefore widely used to generate alginate-based particles for sustained delivery systems [20]. In aquaculture, alginate has been explored mainly for oral vaccination platforms [11,22,24] and has also been incorporated into injectable formulations [43,44], supporting its versatility across routes of administration.

Morphological analysis by TEM revealed a clear electron-density contrast between the core and the surrounding layer in VP2-loaded particles (Figure 5C), a feature previously reported as compatible with successful biomolecule encapsulation [45]. In addition to morphology, particle size distribution and surface charge are critical attributes that can influence colloidal stability, antigen release kinetics, cellular uptake, and ultimately immunogenicity [45,46]. In our study, TEM indicated particle sizes predominantly within ~500–800 nm for VP2-loaded particles, while empty particles were larger (~1000–1500 nm) (Figure 5C,D). This size range is immunologically relevant: particles spanning several hundred nanometers up to a few micrometers can be preferentially taken up by phagocytosis and/or macropinocytosis, promoting antigen presentation and humoral responses [47]. Moreover, the observed spherical morphology may favor internalization compared with high-aspect-ratio particles, although the literature reports some variability depending on the cellular context and particle properties [48,49].

A marked difference was observed between particle sizes determined by TEM and DLS, with the latter yielding substantially larger hydrodynamic diameters. This discrepancy has been widely described for alginate-based particulate systems and reflects the different principles underlying the two techniques. DLS measures the hydrodynamic diameter of particles in suspension and, when reported as an intensity-weighted distribution, is particularly sensitive to the presence of aggregates because light scattering increases approximately with the sixth power of particle diameter. Consequently, even a relatively small proportion of aggregates can disproportionately increase the apparent particle size measured by DLS [43]. In contrast, TEM provides direct visualization of dehydrated individual particles. Consistent with this interpretation, both TEM and SEM revealed the presence of particle aggregates (Figure 5C–F), which likely contributed to the larger hydrodynamic diameters observed by DLS. Although aggregation was qualitatively identified, it was not quantitatively assessed, nor was its influence on antigen release kinetics or cellular uptake evaluated. Therefore, these aspects should be considered when interpreting the physicochemical characterization and warrant further investigation in future studies.

The PDI values obtained (below ~0.6; Figure 5A,B) suggest moderate heterogeneity. While some studies report lower PDIs in alginate-based systems, PDIs can vary substantially depending on formulation parameters [22,50]. Importantly, heterogeneity in size distribution may translate into differences in uptake pathways and adjuvant-like behavior, as broader size ranges can engage distinct mechanisms of antigen processing [47]. Thus, further narrowing the size distribution could be beneficial for manufacturing consistency and for mechanistic interpretation.

Zeta potential values were moderately negative (−17.3 ± 0.6 mV for VP2-loaded particles; −24.6 ± 0.5 mV for empty particles). These values are consistent with alginate surfaces dominated by deprotonated carboxyl groups and typically reflect moderate electrostatic stabilization [51,52]. The lower negative value for VP2-loaded particles suggests that protein–polymer interactions can partially mask surface charge, consistent with previous reports [53]. Although zeta values did not reach the ±30 mV often associated with high colloidal stability, a moderately negative charge may represent a favorable balance between stability and biocompatibility. Strong positive charges can enhance uptake but may increase membrane disruption and cytotoxicity, whereas moderate negative charges often reduce toxicity risks [54]. Overall, the physicochemical profile supports the feasibility of the formulation as an antigen delivery platform and indicates clear avenues for optimization (e.g., reducing aggregation and improving colloidal stability).

Encapsulation efficiency (EE) and loading capacity (LC) are key formulation attributes because they influence antigen protection, dose control, and the release profile [55,56]. In this study, EE ranged from ~41–47%, and LC was ~13% (Table 2). While EE values were lower than those reported in some alginate-protein systems [11,49,57], LC values were within previously reported ranges [48,56]. Differences in EE may reflect formulation-specific factors such as alginate concentration, crosslinking kinetics, mixing/shear conditions, and the physicochemical properties of the antigen. Studies suggest that increasing alginate concentration, altering the gelation rate, or incorporating suitable additives can improve encapsulation performance [58]. Importantly, SDS-PAGE analysis of released VP2 indicated preserved integrity (Figure 6), supporting that the encapsulation and release process did not substantially compromise antigen stability.

Despite the favorable physicochemical characteristics observed, some limitations remain, including moderate particle heterogeneity, aggregation, and limited colloidal stability, which could influence antigen uptake and immune responses. In addition, encapsulation efficiency may still be improved to optimize antigen retention and release control. Future studies should therefore focus on refining formulation parameters such as alginate concentration, crosslinking conditions, and stabilization strategies to reduce aggregation, improve particle homogeneity, and enhance antigen stability and delivery efficiency.

### 4.3. Immune Response After Immersion Vaccination: Humoral and Early Antiviral Signatures

Immersion vaccination is widely used in early life stages of fish due to its practicality and suitability for mass immunization [59,60]. In this context, both humoral and transcriptional responses were evaluated to characterize the immune profile induced by the VP2-MP formulation. In teleost, IgM is the main systemic antibody, and the anterior kidney is a key organ for both innate and adaptive immune responses [61,62].

The serological results showed that VP2-MP induced increased levels of specific IgM at 18 and 30 days post-immunization, with values higher than those in the IPNV and PBS controls across all serum dilutions. Similar responses have been partially reported for immersion vaccines, including microparticle-based formulations, where antibody levels increase after 2–3 weeks depending on environmental conditions such as temperature, [63,64]. Although this pattern was maintained at higher dilutions, differences were not always statistically significant [65]. The stabilization of antibody levels between 18 and 30 days is consistent with the typical dynamics of humoral responses in fish [63]. This response may be influenced by the physicochemical properties of the microparticle system, including particle size and surface charge, which can facilitate antigen uptake and sustained release [45,46,47]. Given the preliminary nature of this study, future work should include the evaluation of neutralizing antibody titers using the IPNV infection model in CHSE-214 cells, in order to further characterize the functional relevance of the antibody response induced by vaccination.

Inter-individual variability was observed, particularly within the VP2-MP group, where subpopulations of high and moderate responders were detected. Such variability has been widely reported in salmonids and reflects inherent differences in immune responsiveness [66,67]. Although alginate-based microparticles may contribute to a degree of non-specific immune activation, their overall immunogenic contribution is considered low [21,68], suggesting that the observed response is mainly directed against the VP2 antigen.

At the transcriptional level, VP2-MP induced a distinct immune profile compared to IPNV. While IPNV triggered high fold-change values in several genes, these responses were associated with marked variability and a delayed peak. This is consistent with previous reports describing strong interferon-mediated responses induced by inactivated viral vaccines containing multiple pathogen-associated molecular patterns (PAMPs) [69]. However, such responses may not necessarily reflect more efficient immune regulation [70,71].

VP2-MP, however, was characterized by moderate and more homogeneous expression levels, with earlier activation at 24 h. This pattern was observed for both innate (ifn-α, mx) and adaptive (t-bet, ifn-γ) markers, suggesting a more balanced temporal response [71,72]. The coordinated expression of t-bet and ifn-γ supports activation of a Th1-like pathway, which appeared more consistent in VP2-MP-treated fish [73,74].

These findings are consistent with evidence suggesting that moderate, regulated gene expression responses may be more biologically relevant than highly variable, high-magnitude responses [75]. The integration of serological and transcriptional data supports this interpretation, as VP2-MP induced higher antibody levels across dilutions, suggesting an effective humoral response [76,77]. In contrast, the stronger but more variable transcriptional response observed in IPNV was not associated with higher antibody levels, suggesting differences in how these responses are translated into humoral immunity.

Despite these findings, some limitations should be considered. The relatively small sample size and the restricted sampling period may have influenced the detection of significant differences and limited conclusions on response duration. Additionally, the limited panel of immune markers evaluated provides only a partial view of the immune response elicited by VP2-MP, highlighting the need for further mechanistic studies to better characterize the pathways involved.

Taken together, VP2-MP showed concordant responses across serological and transcriptional analyses, characterized by detectable antibody responses and moderate modulation of immune-related gene expression levels. These findings support the potential of VP2-MP as an immersion-based vaccine platform for inducing immune responses in salmonids. However, further optimization of the formulation and additional studies, including challenge experiments, are required to evaluate its protective capacity against IPNV.

### 4.4. Limitations and Perspectives for Optimization

While the formulation demonstrated immunogenicity following immersion and induced early antiviral signatures, several aspects may benefit from further optimization. The moderate zeta potential and imaging evidence of aggregation suggest that improving colloidal stability could enhance formulation consistency and potentially favor antigen delivery to mucosal immune sites. In addition, the encapsulation efficiency (EE) was acceptable but lower than that reported for some alginate–protein systems, indicating that formulation parameters (e.g., polymer concentration, crosslinker addition rate, emulsification conditions, or excipients) could be further optimized to improve antigen retention and dose efficiency [58]. Furthermore, limitations associated with recombinant VP2 production, including possible partial solubilization and protein aggregation during bacterial expression, may have affected antigen recovery and structural integrity, potentially influencing encapsulation performance and immunogenicity.

Although the immunological data suggest the induction of humoral and antiviral responses, several limitations of the present study should be considered. Following vaccination, each experimental treatment (VP2-MP, Al-MP, IPNV and PBS) was maintained in a separate tank; however, the biological replicates corresponding to each treatment were housed within the same tank. Consequently, potential tank effects cannot be completely excluded because fish within each treatment experienced identical environmental conditions throughout the experimental period. Future studies incorporating independent replicate tanks for each treatment would strengthen the experimental design and the statistical independence of biological replicates. In addition, the relatively small sample size and the restricted sampling period may have influenced the detection of significant differences and limit conclusions regarding the duration of the immune response. Moreover, the analysis focused on a limited number of immune markers; therefore, more comprehensive studies will be required to better assess the efficacy of the vaccine prototype. In addition, protective efficacy remains to be confirmed through challenge studies and by evaluating mucosal immunity, including local antibody responses and tissue-specific antiviral markers.

## 5. Conclusions

In this study, a vaccine platform based on alginate microparticles loaded with recombinant VP2 protein (VP2-MP) was developed and evaluated in *Salmo salar* following immersion immunization. The formulation induced detectable IgM responses and modulated the expression of antiviral and immune-related genes, including ifn-α, mx, t-bet, and ifn-γ.

Compared to the inactivated virus formulation, VP2-MP was associated with a more moderate and less variable transcriptional response, together with higher antibody levels across serum dilutions. This suggests differences in how immune responses are induced and maintained between formulations.

Collectively, these results indicate that vaccine platforms such as the one developed in this study, based on alginate microparticles loaded with recombinant IPNV VP2 protein, may represent a promising strategy to improve antigen delivery and immune stimulation in immersion vaccines for salmonids. However, further studies are required, including optimization of the formulation and evaluation of different antigen doses. In addition, longer sampling periods, broader immunological characterization, and challenge trials are necessary to confirm protective efficacy.

## Figures and Tables

**Table 2:** EE and LC values for three VP2-MP batches.

Batch	Initial Protein Concentration (mg/mL)	Final Protein Concentration (mg/mL)	EE (%)	LC (%)
1	0.46	0.20	43.5	13.0
2	0.46	0.19	41.3	12.9
3	0.49	0.23	46.9	13.0

## Data Availability

The original contributions presented in this study are included in the article. Further inquiries can be directed to the corresponding author.

## Appendix A

**Table A1:** MALDI-MS/MS identification of recombinant VP2 protein. Identified tryptic peptides mapped across the VP2 sequence, resulting in ~38.6% sequence coverage. Peptides are classified according to confidence level and annotated for common modifications, including methionine oxidation and cysteine carbamidomethylation.

Peptide No.	Sequence	m/z (Da)	Position (aa)	Score	Modification
1	WNANQTGLEFDQWLETSQDLK	2523.503	70–90	1033	—
2	QETSSYNLEVSESGSGILVCFPGAPGSR	2928.727	36–63	144	Carbamidomethyl (C)
3	GVTVLNLPTGFDKPYVR	1876.264	160–176	—	—
4	SIMLPETGPASIPDDITER	2042.274	13–31	—	—
5	SIMLPETGPASIPDDITER	2058.294	13–31	—	Oxidation (M)
6	FDFQLDFMGLDNDVPVVTVVSSVLATNDNYR	3490.983	256–286	—	—
7	GIRKVAAPVLSTLFPMAAPLIGMADQFIGDLTK	3458.033	451–483	—	Oxidation (M)
8	IGAHYR	716.512	64–69	—	—
9	MNTNK	607.418	1–5	—	—
10	MNTNK	623.433	1–5	—	Oxidation (M)
11	MRCTAAIAPRRYEIDLPSQR	2420.278	193–212	—	Oxidation (M); Carbamidomethyl (C)
12	MILSHREELDIRTVWR	2070.291	401–416	—	Oxidation (M)
